# Supplementary material for: Polymyxin B Peptide Hydrogel Coating: A Novel Approach to Prevent Ventilator-Associated Pneumonia
Source: Int J Mol Sci. 2024 Sep 24;25(19):10269. doi: 10.3390/ijms251910269 (PMC11477085; doi:10.3390/ijms251910269)
Supplement: Supplementary file 1 [file ijms-25-10269-s001.zip › ijms-3160422-supplementary.pdf]

Supporting Materials
For

Polymyxin B peptide hydrogel coating: a novel approach to prevent ventilator-associated pneumonia

Milan Wouters [1,†], Laurence Van Moll [1,†], Linda De Vooght [1], Emilia Choińska [2], Joanna Idaszek [2], Karol Szlązak [2], Marcin K. Heljak [2], Wojciech Święszkowski [2,*] and Paul Cos [1,*]

Table of contents

S1. Subtracted spectra 1

S2. Raman spectroscopy 2

S3. Wettability 4

S4. Cytocompatibility 5

S5. Computer code compression mechanical analysis 6

S1. Subtracted spectra

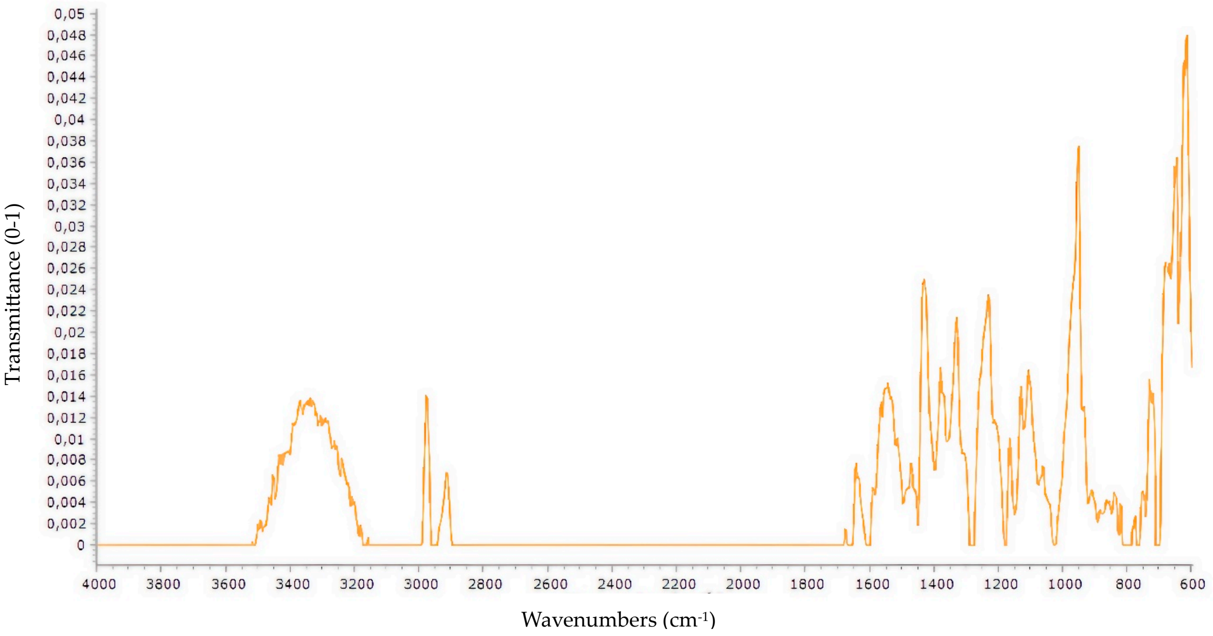

Figure S1a: Subtraction transmittance FTIR spectrum of the DMAPS_AA_PVC spectrum subtracted by the DMAPS_AA_PMB_PVC spectrum.

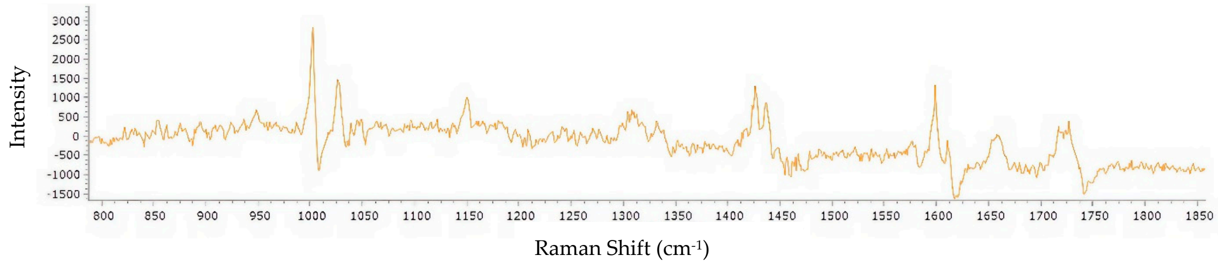

Figure S1b: Subtraction intensity Raman spectrum of the DMAPS_AA_PMB_PVC spectrum subtracted by the DMAPS_AA _PVC spectrum.

S2. Raman spectroscopy

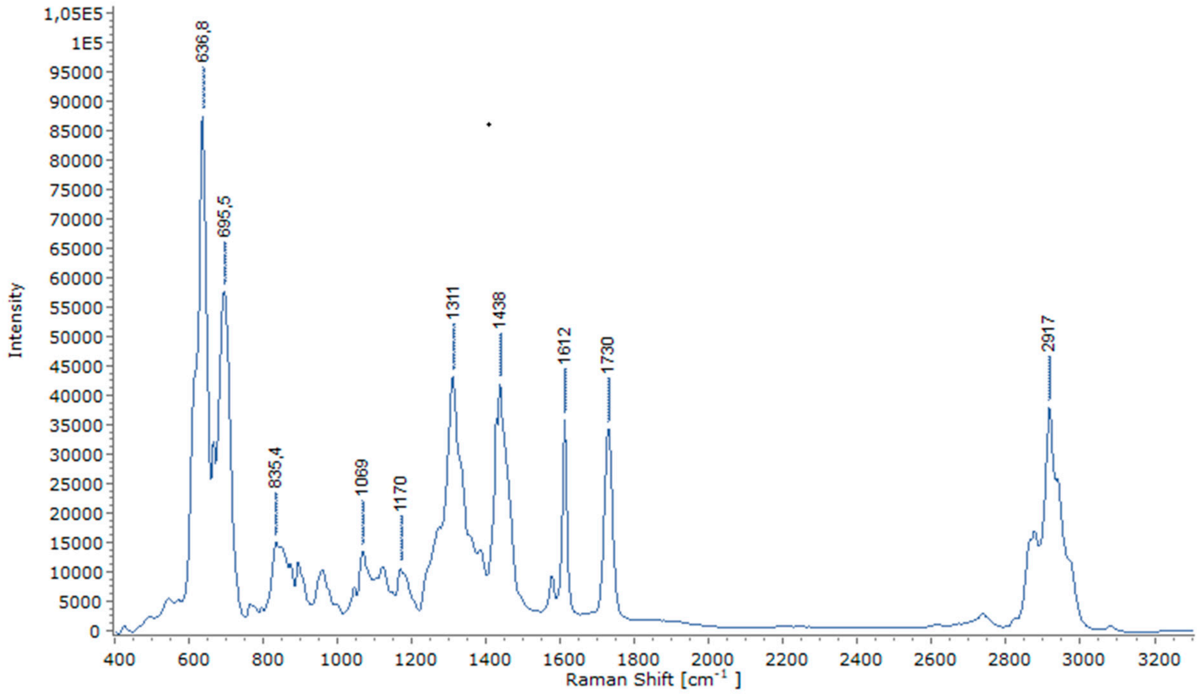

Figure S2a: Extended Raman spectrum of uncoated PVC.

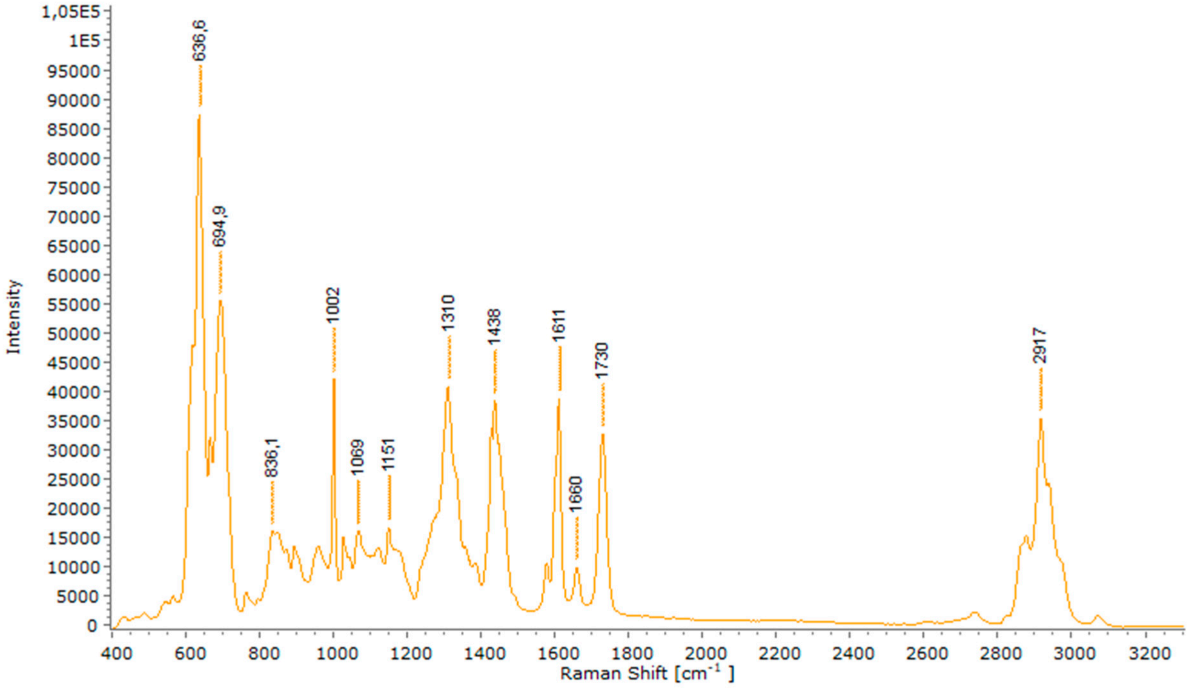

Figure S2b: Extended Raman spectrum of DMAPS_AA_PVC.

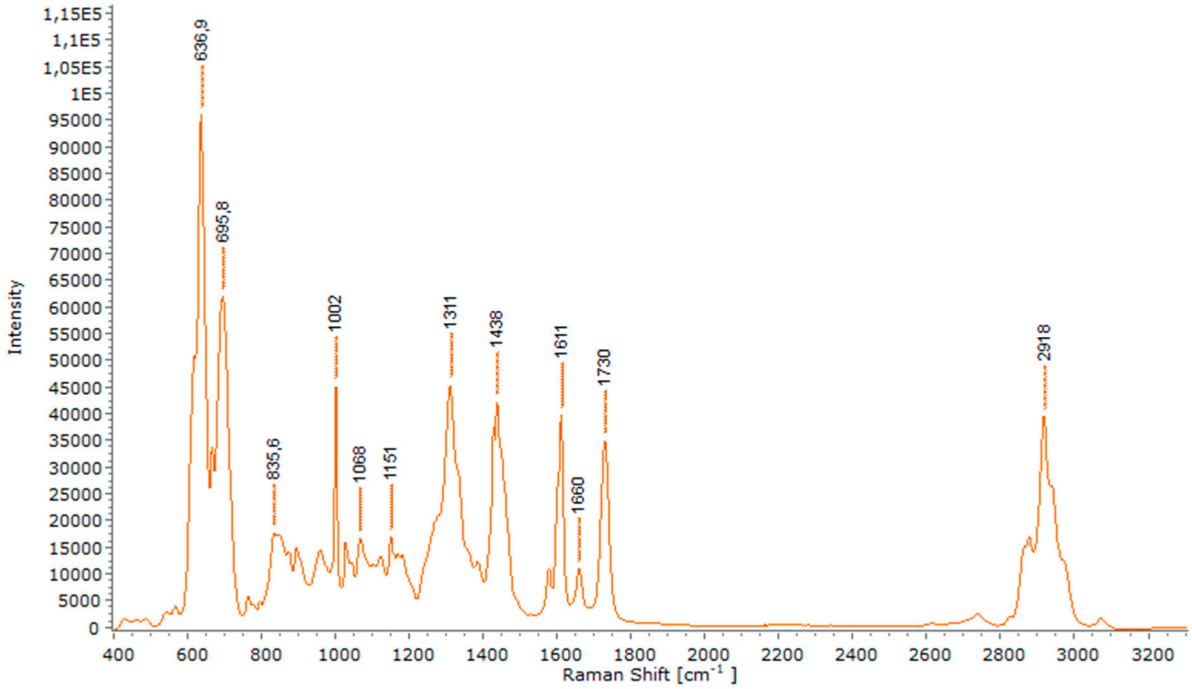

Figure S2c: Extended Raman spectrum of DMAPS_AA_PMB_PVC.

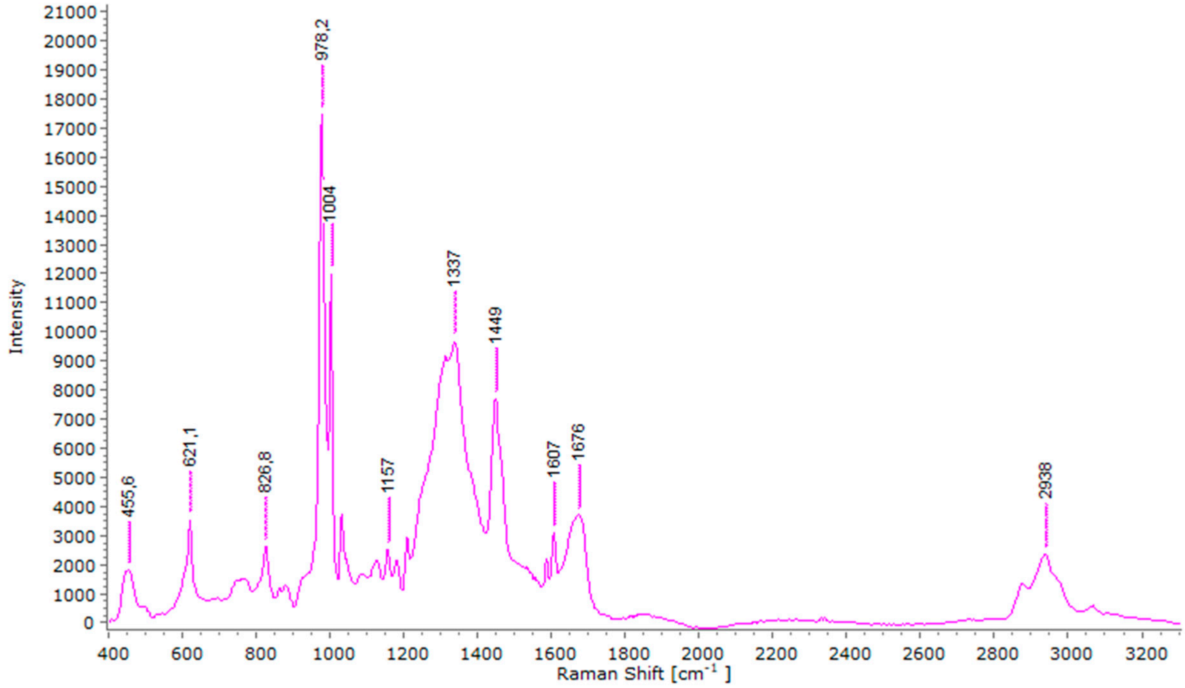

Figure S2d: Extended Raman spectrum of polymyxin B.

S3. Wettability

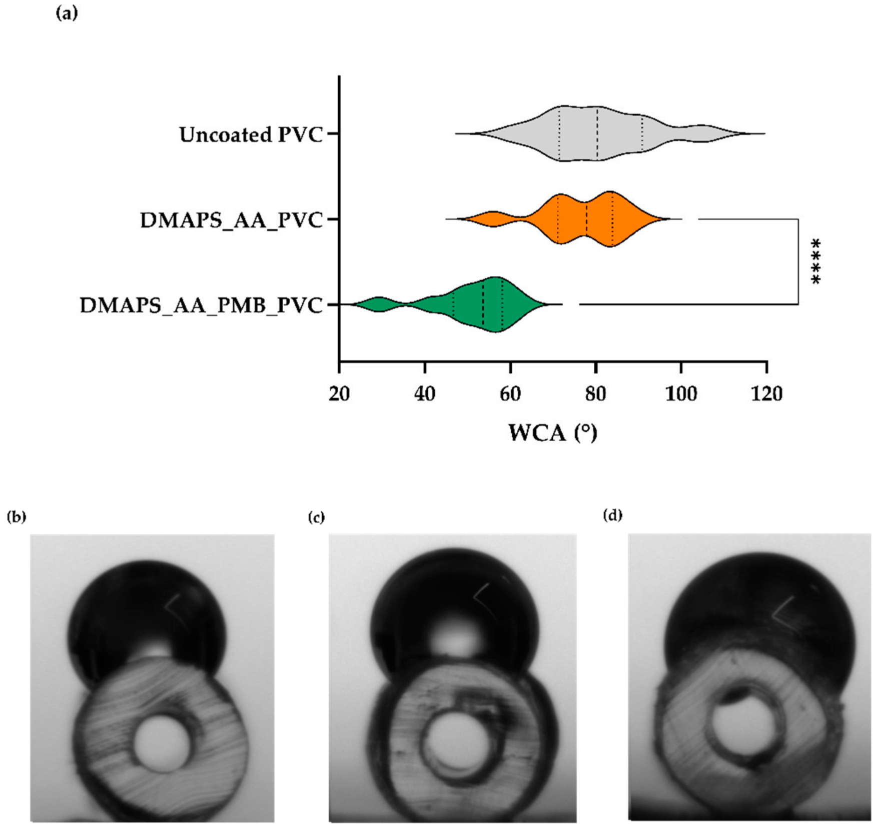

Figure S3: Frontal water contact angle. (a) Violin-plot of the water contact angle (WCA) calculated as median (full vertical line) of 10 measurements ± the standard deviation. Dotted lines represent the first and third quartile. An unpaired t test with Welch correction was used to test for statistical difference ($p \leq 0.05$). **(b-d)** Water contact angle image of **(b)** uncoated PVC **(c)** DMAPS_AA_PVC and (d) DMAPS_AA_PMB_PVC

S4. Cytocompatibility

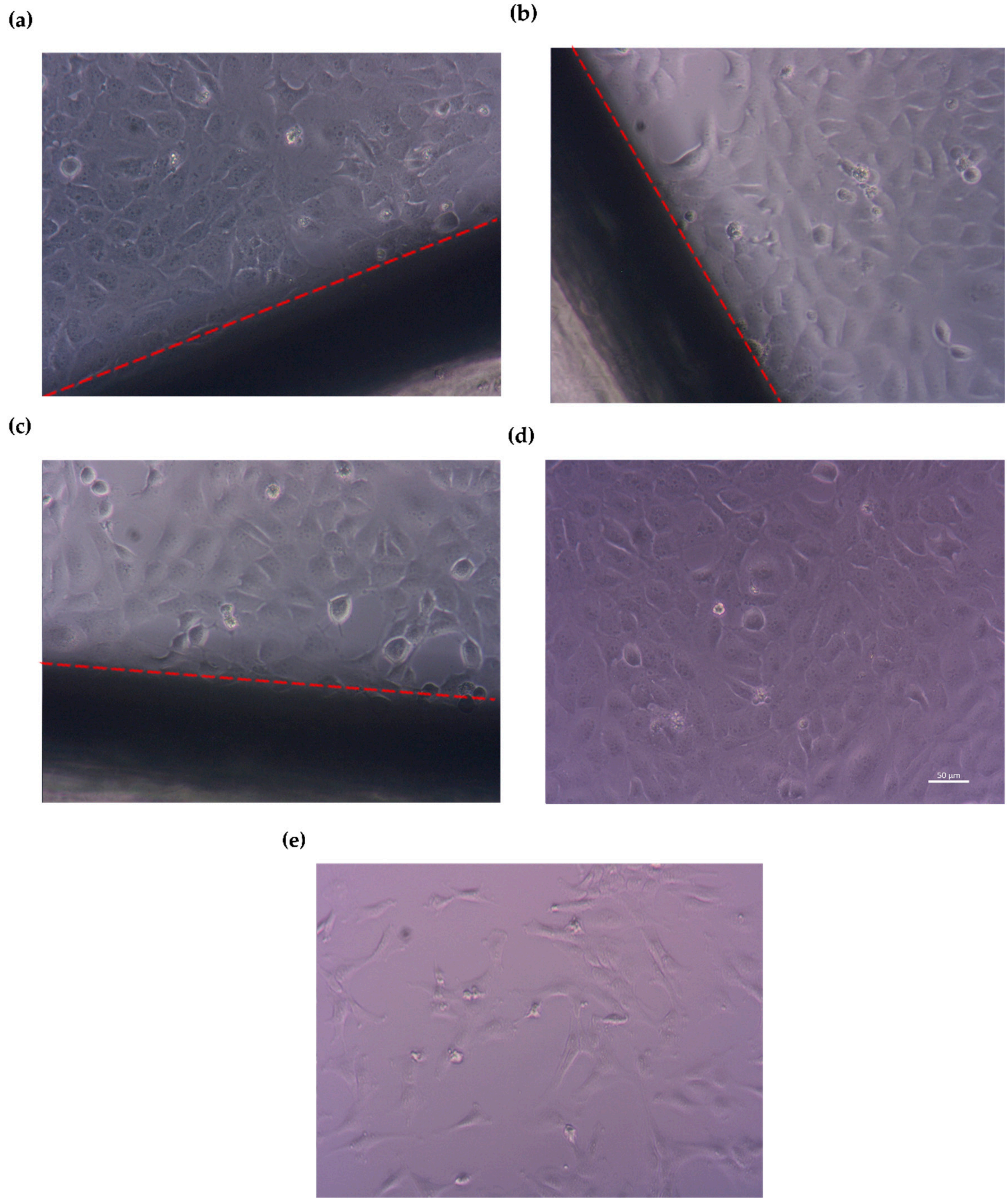

Figure S4: Microscopic evaluation of the morphology of MRC5-SV2 fibroblasts cultured in the presence of **(a)** uncoated PVC, **(b)** DMAPS_AA_PVC, **(c)** DMAPS_AA_PMB_PVC, **(d)** negative control and **(e)** positive control. The red line in figure S4a-c indicates the border between the tubing and the cells.

S5. Computer code compression mechanical analysis

Table S1a: Code used for plotting the mechanical properties. Strain/Force1-3 represented uncoated PVC, strain/force4-6 represented DMAPS_AA_PVC and strain/force7-9 represented DMAPS_AA_PMB_PVC.

```
import numpy as np

import matplotlib.pyplot as plt


# Data

Strain1 = np.array([ ])  # Replace with actual data

Force1 = np.array([ ])   # Replace with actual data


Strain2 = np.array([ ])  # Replace with actual data

Force2 = np.array([ ])   # Replace with actual data


Strain3 = np.array([ ])  # Replace with actual data

Force3 = np.array([ ])   # Replace with actual data


Strain4 = np.array([ ])  # Replace with actual data

Force4 = np.array([ ])   # Replace with actual data


Strain5 = np.array([ ])  # Replace with actual data

Force5 = np.array([ ])   # Replace with actual data


Strain6 = np.array([ ])  # Replace with actual data

Force6 = np.array([ ])   # Replace with actual data


Strain7 = np.array([ ])  # Replace with actual data

Force7 = np.array([ ])   # Replace with actual data


Strain8 = np.array([ ])  # Replace with actual data

Force8 = np.array([ ])   # Replace with actual data


Strain9 = np.array([ ])  # Replace with actual data

Force9 = np.array([ ])   # Replace with actual data
```

```
# Interpolate data to a common strain axis
common_strain = np.linspace(0, 30, 193)
hs_strain = np.linspace(0, 30, 193)
pmb_strain = np.linspace(0, 30, 193)

Force1_interp = np.interp(common_strain, Strain1, Force1)
Force2_interp = np.interp(common_strain, Strain2, Force2)
Force3_interp = np.interp(common_strain, Strain3, Force3)
Force4_interp = np.interp(hs_strain, Strain4, Force4)
Force5_interp = np.interp(hs_strain, Strain5, Force5)
Force6_interp = np.interp(hs_strain, Strain6, Force6)
Force7_interp = np.interp(pmb_strain, Strain7, Force7)
Force8_interp = np.interp(pmb_strain, Strain8, Force8)
Force9_interp = np.interp(pmb_strain, Strain9, Force9)

# Calculate mean and standard deviation
mean_force1 = np.mean([Force1_interp, Force2_interp, Force3_interp], axis=0)
std_force1 = np.std([Force1_interp, Force2_interp, Force3_interp], axis=0)
mean_force2 = np.mean([Force4_interp, Force5_interp, Force6_interp], axis=0)
std_force2 = np.std([Force4_interp, Force5_interp, Force6_interp], axis=0)
mean_force3 = np.mean([Force7_interp, Force8_interp, Force9_interp], axis=0)
std_force3 = np.std([Force7_interp, Force8_interp, Force9_interp], axis=0)

# Plotting
plt.figure(figsize=(10, 6), dpi=300)

# Mean curve
plt.plot(common_strain, mean_force1, color='black', label='Uncoated PVC', linewidth=2)
plt.plot(hs_strain, mean_force2, color='pink', label='DMAPS_AA_PVC', linewidth=2)
plt.plot(pmb_strain, mean_force3, color='green', label='DMAPS_AA_PMB_PVC', linewidth=2)

# Standard deviation shaded area
```

```
plt.fill_between(common_strain, mean_force1 - std_force1, mean_force1 + std_force1, color='gray', alpha=0.3)

plt.fill_between(hs_strain, mean_force2 - std_force2, mean_force2 + std_force2, color='lightpink', alpha=0.3)

plt.fill_between(pmb_strain, mean_force3 - std_force3, mean_force3 + std_force3, color='lightgreen', alpha=0.3)


# Labels and title

plt.xlabel('Strain (%)')

plt.ylabel('Force (N)')

plt.title('DMA compression analysis of coated ETTs')

plt.legend(fontsize=8)

plt.grid(True)

plt.tight_layout()


# Adjusting axis limits

plt.xlim(0, 30)  # Set x-axis limits from 0 to 30

plt.ylim(0, 6)   # Set y-axis limits from 0 to 6


# Show plot

plt.show()
```

Table S1b: Code used for calculation of slope similarity between two graphs. X1 and Y1 representing mean data of uncoated PVC and X2 and Y2 representing average data of either DMAPS_AA_PVC or DMAPS_AA_PMB_PVC.

```
import numpy as np

from scipy.interpolate import interp1d


# Given x and y values for two datasets (replace these with actual data)

x1 = np.array([ ])

y1 = np.array([ ])


x2 = np.array([ ])

y2 = np.array([ ])
```

```
# Define the x range of interest
x_min = 10
x_max = 30

# Define the common x range for interpolation within the specified range
common_x = np.linspace(max(x_min, min(x1[0], x2[0])), min(x_max, max(x1[-1], x2[-1])), 1000)

# Interpolate the datasets using cubic interpolation
interp1 = interp1d(x1, y1, kind='cubic', fill_value="extrapolate")
interp2 = interp1d(x2, y2, kind='cubic', fill_value="extrapolate")

# Compute the interpolated y values for the common x range
y1_interp = interp1(common_x)
y2_interp = interp2(common_x)

# Compute the derivatives (slopes) of the interpolated y values
dy1_dx = np.gradient(y1_interp, common_x)
dy2_dx = np.gradient(y2_interp, common_x)

# Define a tolerance for slope similarity
tolerance = 0.01

# Find x values where slopes are similar within the specified tolerance
similar_slope_indices = np.where(np.abs(dy1_dx - dy2_dx) < tolerance)[0]
similar_slope_x = common_x[similar_slope_indices]
similar_slope_y1 = y1_interp[similar_slope_indices]
similar_slope_y2 = y2_interp[similar_slope_indices]

# Print the x and y coordinates with similar slopes
print("Points with similar slopes:")
for x, y1, y2 in zip(similar_slope_x, similar_slope_y1, similar_slope_y2):
    print(f"x: {x}, y1: {y1}, y2: {y2}")
```
